# Primarily Null Associations Between the Nursing Child Assessment Feeding Scale and Maternal Feeding Behaviors, Infant Eating Behaviors, Diet, and Growth: A Prospective Cohort Study

**DOI:** 10.3390/nu18152532

**Published:** 2026-08-04

**Authors:** Cin Cin Tan, Julie Sturza, Alison K. Ventura, Alison L. Miller, Ashley N. Gearhardt, Niko Kaciroti, Julie C. Lumeng

**Affiliations:** 1 Department of Psychology, University of Toledo, Toledo, OH 43606, USA; cincin.tan@utoledo.edu; 2 Department of Pediatrics, Medical School, University of Michigan, 2800 Plymouth Road, Building 520, Ann Arbor, MI 48109, USA; jmigrin@umich.edu (J.S.); nicola@umich.edu (N.K.); 3 Department of Kinesiology and Public Health, Center for Health Research, Bailey College of Science and Mathematics, California Polytechnic State University, San Luis Obispo, CA 93407, USA; akventur@calpoly.edu; 4 Department of Health Behavior and Health Equity, School of Public Health, University of Michigan, 1415 Washington Heights, Ann Arbor, MI 48109, USA; alimill@umich.edu; 5 Department of Psychology, College of Literature, Science, and the Arts, University of Michigan, 2268 East Hall, Ann Arbor, MI 48109, USA; agearhar@umich.edu

**Keywords:** infant eating, infant feeding, infant growth, anthropometry, sucking, maternal feeding behaviors, maternal feeding practices

## Abstract

**Background/Objectives**: The predictive value of the Nursing Child Assessment Feeding Scale (NCAFS) for eating, diet, and growth outcomes among healthy infants is not well-described. **Methods**: In this prospective cohort study, healthy infants (*n* = 227) were assessed with the NCAFS at 0.5, 2, and 4 months and outcomes were measured concurrently and at 12 months. Maternal feeding and infant eating behaviors were observed during a separate feeding and assessed with the Infant Feeding Styles Questionnaire, Baby Eating Behavior Questionnaire, Children’s Eating Behavior Questionnaire-Toddler, and a diet questionnaire. Sucking was measured and anthropometry obtained. **Results**: In mixed models with repeated measures, concurrent associations included maternal verbalization while waiting for a separate feeding with maternal Sensitivity to Cues (β = 0.80) and maternal Cognitive Growth Fostering (β = 1.05); maternal verbalization during a separate feeding with maternal Social–Emotional Growth Fostering (β = 1.10) and maternal Cognitive Growth Fostering (β = 1.37); suck duration with infant Clarity of Cues (β = −5.35); feeding duration with maternal Social–Emotional Growth Fostering (β = 0.04), maternal Cognitive Growth Fostering (β = 0.03), and infant Responsiveness to Caregiver (β = 0.06); and bottle-feeding intensity with maternal Social–Emotional Growth Fostering (β = −1.11). Prospective associations included maternal Sensitivity to Cues with infant weight-for-length z-score change per month (β = −0.02) and maternal Social–Emotional Growth Fostering with breastmilk feeding intensity (β = 0.06). **Conclusions**: Associations were primarily null. However, more social interaction during feeding, less nutritive sucking (i.e., shorter suck duration), longer feeding duration, and lower bottle-feeding intensity were associated with more optimal NCAFS scores concurrently, and more optimal NCAFS scores were associated with lower weight-for-length z-score change and greater breastmilk feeding intensity prospectively.

## 1. Introduction

Feeding has long been an important context for evaluating mother–infant interaction, dating back to Ainsworth’s 1967 description of maternal sensitivity and synchrony [1]. In 1970, the United States Public Health Service requested development of a screening tool to identify infants at risk for future health and developmental difficulties based on the caregiving environment. Through collaboration among clinicians and researchers in developmental psychology, psychobiology, nursing, and observational methods [2,3,4], the resulting Nursing Child Assessment Satellite Training enabled assessment of high-quality mother–child interaction in either a structured teaching or naturalistic feeding context [5]. The tool for the naturalistic feeding context is the Nursing Child Assessment Feeding Scale (NCAFS). The Barnard Child Health Assessment Model [6] defines key adaptive elements for the mother—including sensitivity to infant cues, alleviation of distress, and fostering social–emotional and cognitive growth—and for the infant—including clear cues and responsiveness to the caregiver. With the goal of translating research into practice, the tools were simplified in 1975 for application by community public health nurses [4,7] and, after minor modifications in 1994, they are now also known as the Parent–Child Interaction Scales [7]. The scales are the most widely used standardized measures of mother–infant interaction quality and continue to be used in clinical practice and research today [8,9].

With the emergence of the childhood obesity epidemic [10], research interest in maternal sensitivity as a possible contributor emerged [11]. By the 2010s, it became clearer that childhood obesity has its origins before the age of two years [12], and researchers and interventionists increasingly focused on the quality of the feeding interaction [13]. An optimal feeding interaction is hypothesized to prevent overfeeding through attention to infant hunger and satiety cues [14,15]. As research in this area has grown, methodological limitations have become more apparent. In particular, there is not a full consensus on the definition or operationalization of optimal maternal–infant interaction in the feeding context [14,16,17]. Further, the most relevant constructs are especially prone to bias when measured by maternal self-report, underscoring the need for observational measures. Measuring observed behavior during feeding is complex, often requiring substantial expertise and training [18]. The NCAFS has therefore appealed to researchers, given that it is well-recognized, has been more extensively evaluated psychometrically than many other tools [19,20,21], has a normative database [2], can be applied to naturalistic feeding of breast, bottle, or solids lasting less than 30 min [9], can be used from birth to 12 months, and can be learned in a several-day workshop [2].

The NCAFS was originally designed to measure the quality of maternal–infant interaction using the feeding interaction as one of two contexts. Since its development more than fifty years ago, a large body of research has emerged focused on detailed analysis of various elements of the maternal–infant feeding interaction and how these may contribute to infant growth and eating behaviors. Many of the newer tools used by researchers in the field expand on constructs included in the NCAFS (e.g., responding to the infant’s hunger and satiety cues). However, the NCAFS also includes many items that may be less relevant to—and even counter to—the infant’s ability to regulate energy intake. Specifically, mothers receive more optimal scores for being more interactive with the infant and varying their intensity of interaction. For at least some infants, however, greater frequency and/or intensity of interaction with the feeder may interfere with their ability to accurately regulate energy intake—increasing the risk of both over- [22] and under- [23,24] eating.

Few studies have examined NCAFS scores in relation to infant eating behavior, dietary intake, and weight gain. NCAFS subscales have been shown to have positive [25], negative [26,27], and null [28] associations with infant weight. Mothers distracted during bottle-feeding showed lower sensitivity on the NCAFS and their infants consumed more during the observed feeding [29]. NCAFS subscales have been positively associated with breastfeeding initiation and continuation [30,31,32,33]. Therefore, this study sought to evaluate, in a cohort of healthy infants using a within-subject repeated measures design across ages 0.5, 2, and 4 months, how each of the six NCAFS subscales associates with (1) concurrent maternal feeding behaviors measured by observation in an alternative feeding task and by maternal report; (2) concurrent infant eating behaviors measured by observation in an alternative feeding task, by maternal report, and indexed by sucking patterns and feeding duration; (3) concurrent infant habitual dietary intake; (4) concurrent infant anthropometry; and (5) infant eating behavior, habitual diet, and anthropometry at 12 months.

## 2. Materials and Methods

### 2.1. Overview

The overall study sought to examine the development of infant eating behavior longitudinally within participants at ages 0.5, 2, 4, 6, 9, and 12 months based on data collected from questionnaires, videotaped structured tasks, and anthropometry. Trained research staff collected data via home visits. Staff followed detailed manuals of procedures. Fidelity to the study protocol was checked at regular intervals through review of videotapes. Study data were collected and managed using REDCap v9.3, a secure, web-based platform that provided standardization of data collection and entry, including programmed field types and validation rules. Research staff and participating mothers were blind to study hypotheses. The study was approved by the University of Michigan institutional review board (HUM00103575). Mothers provided written informed consent.

### 2.2. Participants and Recruitment

A convenience sample was recruited through flyers, social media, and targeted phone, email, and mail outreach to pregnant women and mothers of newborns living within 1.5 h of Ann Arbor, Michigan. The study was described as examining infant eating behavior and mother–infant interactions in the first year after birth, with data collection planned at ages 0.5, 2, 4, 6, 9, and 12 months. Infants were eligible to enroll at ages 0.5, 2, or 4 months. Inclusion criteria were gestational age of 37.0 to 42.0 weeks, weight appropriate for gestational age, no significant perinatal or neonatal complications, the biological mother being a legal and custodial guardian, and the infant consuming two ounces or more from an artificial nipple at least once weekly. Exclusion criteria were maternal age younger than 18 years or non-English speaking, infant medical conditions affecting eating, growth, or development, or child protective services involvement. This analysis focused on the feeding interaction at ages 0.5, 2, and 4 months because feeding during this developmental period remains exclusively milk- or formula-feeding and we posited that the introduction of solids to the diet could introduce significant additional variability.

### 2.3. Feeding Tasks

#### 2.3.1. Typical Feeding

At 0.5, 2, and 4 months, mothers were videotaped offering a typical bottle-feeding. Mothers prepared breastmilk or usual formula in their usual manner with one additional ounce added to avoid constraining intake, and feedings began when mothers indicated infants were hungry. Mothers were asked to feed as usual and end the feeding when they thought infants were finished. During the typical feeding, research staff recorded bottle content, feeding duration, and bottle weight measured in duplicate before and after feeding using a calibrated scale (precision ±0.5 g, TE32FT Digital Scale; Taylor Precision Products Inc., Oak Brook, IL, USA) and averaged, from which intake was calculated. Time since last feeding was tested as a covariate and was not associated with any NCAFS subscale and was not considered further.

#### 2.3.2. Ability to Delay Gratification for Food in Infants Task

At 0.5, 2, and 4 months, dyads completed the Ability to Delay Gratification for Food in Infants Task (ATDG-FIT) [34], which assesses infant behavior when required to wait for feeding. When mothers indicated hunger, they held the infant as usual while the bottle or breast was visible but withheld for 3 min. Mothers were told they could interact with the infant however they wished but were not permitted to feed the infant. This 3 min period is referred to as the “delay segment.” Following an additional 2 min delay during which a pacifier could also be offered, mothers were told that they could feed the infant. No other instructions regarding the interaction during feeding were provided. The first 1 min period of feeding is referred to as the “feeding segment.” The entire protocol was videotaped.

### 2.4. Behavioral Coding

#### 2.4.1. NCAFS Behavioral Coding

Videotapes of the typical feedings were coded using the NCAFS [5,6] by coders trained to 90% reliability by a certified trainer [6] and 20% of videos were double-coded. The NCAFS comprises 76 items coded as present (=1) or absent (=0), yielding six summed subscales with higher scores representing more of the construct. Sensitivity to Cues assesses caregivers’ ability to recognize and respond to infants’ feeding cues (inter-rater reliability κ = 0.70 to 0.77, 16 items, internal reliability α = 0.37 to 0.55). Response to Child’s Distress assesses caregivers’ ability to soothe or quiet a distressed infant (κ = 0.74 to 0.88, 11 items, α = 0.63 to 0.70). Social–Emotional Growth Fostering (κ = 0.78 to 0.84, 14 items, α = 0.55 to 0.67) and Cognitive Growth Fostering (κ = 0.63 to 0.83, 9 items, α = 0.65 to 0.73) assess caregivers’ ability to provide experiences and social–emotional/cognitive stimulation that facilitate and scaffold the infant’s growth and development. Clarity of Cues assesses infants’ ability to send clear cues to the caregiver (κ = 0.72 to 0.88, 15 items, α = 0.48 to 0.63), and Responsiveness to Caregiver assesses infants’ ability to respond to the caregiver’s attempts to communicate and interact (κ = 0.72 to 0.84, 11 items, α = 0.54 to 0.62). Infant state at feeding initiation was coded ordinally as Quiet sleep (=1), Active sleep (=2), Drowsy (=3), Quiet alert (=4), Active alert (=5), and Crying (=6).

#### 2.4.2. Maternal Feeding Behaviors and Infant Hunger Cues Coded from ATDG-FIT

Behaviors were coded as present or absent for each 10 s interval within each ATDG-FIT segment. Coders were trained to reliability and 20% of videos were double-coded. Maternal verbal communication (e.g., talking, whispering, singing) was coded in delay and feeding segments; inter-rater reliability ranged from κ = 0.83 to 0.84 across age points. The proportion of each ATDG-FIT segment with any maternal verbal communication was calculated. Infant gaze to bottle [35] or fingers-to-mouth [36] behaviors were treated as hunger cues. For bottle-feeding dyads, infant gaze toward the bottle and contact of fingers or hand to the mouth were coded in the delay segments; inter-rater reliability ranged from κ = 0.82 to 0.93 for gaze and 0.69 to 0.72 for fingers to mouth. The proportions of the ATDG-FIT delay segment for which the infant exhibited gaze to bottle or fingers to mouth were calculated.

### 2.5. Sucking Measurement

Sucking was measured as an indicator of hunger and on the premise that mothers may alter their feeding behavior based on the infant’s sucking. Sucking was measured during the typical feeding task in a subset of infants (56% at 0.5 mo, 57% at 2 mo, and 59% at 4 mo) using the NFANT™ Feeding Solution (NFANT™ Labs, Atlanta, GA, USA) [37,38], which is an instrumented bottle with a cantilever sensor and pressure transducer to derive sucking metrics. To balance nipple familiarity with standardization, either a Dr. Brown’s™ or NFANT™ standard nipple was used, selected to approximate the infant’s typical flow rate (9, 15, or 30 mL/min). NFANT™ presence was tested as a covariate, was not significantly associated with any NCAFS subscale, and was not considered further in analyses. The resulting pressure wave data were transformed into suck duration, burst duration, and sucking rate, averaged across the feeding [39]. These sucking metrics were selected given that the extent to which sucking is nutritive is positively associated with suck and burst durations and negatively associated with sucking rate [40]. Specifically, when less nutrition is emitted from a nipple, suck and burst durations shorten and sucking rate increases [41,42,43,44,45,46].

### 2.6. Questionnaires

Mothers reported infant birth date, sex, gestational age, birthweight, delivery mode, birth order, maternal birth date, educational attainment, marital status, and pre-pregnancy weight, and household income and size. Maternal and infant race and ethnicity were reported for descriptive purposes. Income-to-needs ratio was calculated as income divided by the federal poverty line for the household size and year. Mothers reported depressive symptoms at infant age of 2 months (0 = not at all to 3 = nearly every day) using the reliable and validated 8-item Patient Health Questionnaire-8 [47]; responses are summed and internal reliability was α = 0.71.

At either 0.5 or 2 months, and again at 4 months, mothers completed an adapted version of the reliable and validated Infant Feeding Styles Questionnaire (IFSQ) [48]. We retained only items referring to current beliefs and behaviors that were developmentally appropriate for 0.5 to 4 months and did not overlap with constructs assessed elsewhere. Responses (1 = disagree or never to 5 = agree or always) were reverse-scored as appropriate and averaged to generate the subscales restrictive (3 items, α = 0.65 and 0.74), which assesses monitoring and limiting the quantity of food consumed, and pressuring (5 items, α = 0.80 and 0.81) which assesses concern with increasing the amount of food the infant consumes.

At each age point, mothers reported the frequency of infant intake of foods from the Infant Feeding Practices Study II questionnaire [49] over the prior 7 days; breastmilk and bottle-feeding intensities were calculated as the proportion of total feedings that were breastmilk [50] and that used an artificial nipple, respectively, and age of introduction of solids was calculated. Mothers reported formula- or milk-feeding behaviors at 0.5, 2, and 4 months (1 = never to 5 = always) using the reliable and validated Baby Eating Behavior Questionnaire (BEBQ) [51]. After reverse scoring as appropriate, responses were averaged to generate the subscales enjoyment of food (4 items, α = 0.61 to 0.70) and food responsiveness (6 items, α = 0.75 to 0.78). Mothers reported infants’ eating behaviors at 12 months (1 = never to 5 = always) using the reliable and validated Children’s Eating Behavior Questionnaire-Toddler (CEBQ-T) [52]. After reverse scoring as appropriate, responses were averaged to generate the subscales food responsiveness (4 items, α = 0.83), satiety responsiveness (5 items, α = 0.71), and food fussiness (6 items, α = 0.84).

### 2.7. Anthropometry

At 0.5, 2, 4, and 12 months, staff trained and certified in the measurement technique measured infant weight and length in duplicate without clothing or a diaper and averaged the values. Weight was measured to the nearest 0.1 kg on a calibrated BD-585 Digital Infant Scale (Tanita, Tokyo, Japan). Length was measured to the nearest 0.01 cm on a pediatric stadiometer (M-PED LB 35-107-X; Ellard Instruments, Monroe, WA, USA). Weight-for-age, length-for-age, and weight-for-length z-scores (WAZ, LAZ, and WLZ) were calculated based on World Health Organization growth standards. Rate of change in WLZ per month between early infancy and age 12 months (ΔWLZ) was calculated as WLZ at 12 months minus WLZ at the last age point measured (0.5, 2, or 4 months), divided by time in months between the two age points. Mothers’ heights were measured with a stadiometer (Seca 217, Hamburg, Germany), pre-pregnancy weight was self-reported, and pre-pregnancy body mass index (BMI) was calculated.

### 2.8. Statistical Analysis

Power analysis for linear mixed models indicates that a sample size of 200 subjects with at least 2 measurements per subject, intraclass correlation of 0.3 or lower, and a Bonferroni adjusted α = 0.008 would be sufficient to detect a medium effect size (f^2^ = 0.10) with more than 85% power. Infants were included in the analytic sample if they had at least one valid NCAFS subscale at one or more age points. The analytic (*n* = 227) and excluded (*n* = 57) samples did not differ across tested characteristics, except that mothers were older in the analytic sample (31.5 vs. 29.9 years, *p* = 0.04) (Supplementary Table S1). Sixty-two infants contributed NCAFS data at all three age points, 105 at two age points, and 60 at one age point.

We used univariate and bivariate analyses to describe cohort characteristics. To address our first four objectives, mixed models with repeated measures were used to test unadjusted associations of maternal feeding behaviors, infant eating behaviors, habitual diet, and anthropometry with concurrent NCAFS subscales. We also tested unadjusted associations of perinatal, demographic, and typical feeding protocol characteristics with concurrent NCAFS subscales for consideration as potential covariates. A Bonferroni correction for multiple comparisons was applied (*α* = 0.05, corrected significance threshold *p* = 0.008). We then fitted adjusted models, including infant birthweight, maternal education, and maternal pre-pregnancy BMI a priori, as well as any additional variables (including those listed in Supplementary Table S3) that met the corrected significance threshold in the unadjusted models. To address our fifth objective, mixed models with repeated measures were used to test associations of each NCAFS subscale with each 12-month outcome (infant eating behaviors, habitual diet, and anthropometry). For infants with multiple NCAFS’s obtained across 0.5, 2, and 4 months, we used the NCAFS at the oldest age point. We controlled a priori for the age at which NCAFS was obtained, infant sex, gestational age, birthweight, birth order, age at introduction of solids, maternal age, maternal education, and maternal pre-pregnancy BMI, given known associations of these characteristics with outcomes [12,51]. A Bonferroni correction for multiple comparisons was applied (*α* = 0.05, corrected significance threshold *p* = 0.008).

## 3. Results

### 3.1. Cohort Characteristics

As shown in Supplementary Table S1, the analytic sample of infants (*n* = 227) was 51% male, 31% first-born, and 62% non-Hispanic white. Mean gestational age was 39.5 weeks, mean birthweight was 3.4 kg, and solids were introduced by a mean age of 6.9 months. For the sample of mothers, 69% had a 4-year college degree or more, 74% were married, and 70% identified as non-Hispanic white. Mean age was 31.5 years, mean pre-pregnancy BMI was 27.8, mean income-to-needs ratio was 3.5, and mean maternal PHQ-8 Total Score was 3.95.

Perinatal and demographic characteristics, NCAFS subscale scores, maternal feeding behaviors, and concurrent and future infant eating behaviors, diet, and anthropometry are shown across age points in Table 1. With regard to maternal NCAFS subscales, variability was modest for Sensitivity to Cues, Response to Child’s Distress, and Social–Emotional Growth Fostering but strong for Cognitive Growth Fostering. Scores averaged above the scale’s median for all subscales, though were more modest for Cognitive Growth Fostering. With regard to infant NCAFS subscales, variability was moderate for Clarity of Cues and Responsiveness to Caregiver and scores averaged well above the scale’s median for Clarity of Cues and at about the scale’s median for Responsiveness to Caregiver.

With regard to maternal feeding behaviors, on average across age points, mothers verbally communicated to infants during 50 to 57% of the ATDG-FIT delay segment and during 19 to 24% of the ATDG-FIT feeding segment; mothers reported moderately restrictive and minimally pressuring feeding. With regard to infant eating behaviors, on average during the ATDG-FIT delay segment, infant gaze was directed to the bottle for 8 to 23% of the time and infants showed fingers-to-mouth behavior 9 to 21% of the time. On average, infants were reported to have high enjoyment of food and moderate food responsiveness. Suck duration averaged 0.65 to 0.69 s, burst duration 22.5 to 60.2 s, and sucking rate 1.5 to 1.6 sucks/sec. Protocol feeding duration averaged 10.9 to 12.8 min. With regard to infant habitual diet, breastmilk feeding intensity averaged 0.68 to 0.75 and bottle-feeding intensity averaged 0.46 to 0.52. With regard to infant anthropometry, infant WLZ averaged 0.00 to 0.15. With regard to the typical feeding protocol characteristics, protocol bottle content was primarily breastmilk, and intake averaged 53.17 to 73.54 g. Most infants (57 to 80%) were in a quiet alert state at protocol feeding initiation.

At 12 months, mothers reported infants to be moderate in food responsiveness (M = 2.54) and satiety responsiveness (M = 2.70), relatively low in food fussiness (M = 2.11), and to have moderate breastmilk feeding intensity (M = 0.33). Infant WLZ averaged 0.42 and rate of change in WLZ between early infancy and age 12 months averaged 0.04 standard deviation units per month.

As shown in Supplementary Table S2, correlations within subscales between age points were modest for maternal Sensitivity to Cues, maternal Response to Distress, infant Clarity of Cues, and infant Responsiveness to Caregiver, ranging in magnitude from 0.01 to 0.28 with a single exception. Correlations between age points for maternal Social–Emotional Growth Fostering and maternal Cognitive Growth Fostering were somewhat stronger, ranging from 0.27 to 0.53. Correlations between NCAFS subscales were highly variable, though maternal Social–Emotional Growth Fostering and maternal Cognitive Growth Fostering showed some of the strongest correlations, ranging from r = 0.69 to 0.77. In contrast, maternal Response to Distress showed little correlation with other subscales, with all r values < 0.40.

### 3.2. Concurrent Associations with NCAFS Subscales

Table 2 shows unadjusted and adjusted mixed models accounting for repeated measures within subjects and with correction for multiple comparisons predicting concurrent NCAFS subscales from maternal feeding behaviors, infant eating behaviors, infant habitual diet, and infant anthropometry. The models predicting concurrent NCAFS subscales from perinatal, demographic, and typical feeding protocol characteristics are shown in Supplementary Table S3. The adjusted models in Table 2 include infant birthweight, maternal education, and maternal pre-pregnancy BMI as well as any additional covariates (including those listed in Supplementary Table S3) that met the Bonferroni-corrected screening criterion (α = 0.05, corrected significance threshold *p* = 0.008). Associations meeting the Bonferroni-corrected screening criterion for significance (α = 0.05, *p* = 0.008) are described below.

#### 3.2.1. Maternal Feeding Behaviors

As shown in Table 2, maternal verbal communication during the ATDG-FIT delay segment was positively associated with maternal Sensitivity to Cues (β = 0.80), and maternal Cognitive Growth Fostering (β = 1.05). Maternal verbal communication during the ATDG-FIT feeding segment was positively associated with maternal Social–Emotional Growth Fostering (β = 1.10) and maternal Cognitive Growth Fostering (β = 1.37). Neither maternal-reported restrictive nor pressuring feeding was associated with any NCAFS subscale.

#### 3.2.2. Infant Eating Behaviors, Habitual Diet, and Anthropometry

As shown in Table 2, displays of hunger cues, including gaze to bottle or fingers-to-mouth behavior during the ATDG-FIT delay segment were not associated with any NCAFS subscale. Neither maternal-reported infant eating behavior, including enjoyment of food nor food responsiveness, was associated with any NCAFS subscale. Of the sucking metrics (suck duration, sucking burst duration, and sucking rate), only suck duration was associated with any NCAFS subscale, showing a negative association with infant Clarity of Cues (β = −5.35). Feeding duration was positively associated with maternal Social–Emotional Growth Fostering (β = 0.04), maternal Cognitive Growth Fostering (β = 0.03) and infant Responsiveness to Caregiver (β = 0.06). There were no associations of breastmilk feeding intensity with any NCAFS subscale. Bottle-feeding intensity was negatively associated with infant Responsiveness to Caregiver (β = −1.11). Infant WLZ was not associated with any NCAFS subscale.

### 3.3. Prospective Associations of NCAFS Subscales in Early Infancy with 12-Month Infant Eating Behavior, Habitual Diet, and Anthropometry

Associations of NCAFS subscales, adjusted for age at which the NCAFS was obtained (0.5, 2, or 4 months), infant sex, gestational age, birthweight, birth order, age at introduction of solids, maternal age, maternal education, and maternal pre-pregnancy BMI, with infant outcomes at 12 months, including food responsiveness, satiety responsiveness, food fussiness, breastmilk feeding intensity, WLZ, and ΔWLZ per month between early infancy and 12 months, are shown in Table 3. Only two of the identified associations met the Bonferroni-corrected screening criterion for significance (α = 0.05, *p* = 0.008). Specifically, maternal Social–Emotional Growth Fostering was positively associated with breastmilk feeding intensity (β = 0.06) and maternal Sensitivity to Cues was negatively associated with ΔWLZ per month between early infancy and 12 months (β = −0.02).

## 4. Discussion

### 4.1. Key Findings

This study had eight key findings. First, maternal verbal communication with the infant during an alternative feeding task was one of the most robust predictors of the NCAFS subscales, including maternal Sensitivity to Cues, maternal Social–Emotional Growth Fostering, and maternal Cognitive Growth Fostering. Second, suck duration was negatively associated with infant Clarity of Cues. Third, feeding duration was positively associated with maternal Social–Emotional Growth Fostering, maternal Cognitive Growth Fostering, and infant Responsiveness to Caregiver. Fourth, bottle-feeding intensity was negatively associated with infant Responsiveness to Caregiver. Fifth, there were no significant associations of maternal self-reported feeding behaviors, observed infant hunger cues, maternal-reported infant eating behaviors, breastmilk feeding intensity, or infant anthropometry concurrently. Sixth, NCAFS subscale scores were not associated with maternal-reported infant eating behaviors or weight-for-length z-score at 12 months. Seventh, maternal Social–Emotional Growth Fostering was positively associated with breastmilk feeding intensity at 12 months. Eighth, maternal Sensitivity to Cues was negatively associated with rate of change in weight-for-length z-score between early infancy and age of 12 months.

### 4.2. Maternal Verbal Interaction During Feeding and Maternal Sensitivity to Cues, Maternal Social–Emotional Growth Fostering, and Maternal Cognitive Growth Fostering

Mothers’ propensity to interact socially with infants during feeding transcended different feeding contexts in this study. Specifically, while verbal communication with infants occurred, on average, during only about half of the period of time when infants were waiting for a feeding and in less than a quarter of the time while actively feeding, verbal interaction observed during this alternative feeding task was one of the most robust predictors of multiple NCAFS subscales, including maternal Sensitivity to Cues, maternal Social–Emotional Growth Fostering, and maternal Cognitive Growth Fostering. This finding aligns with the fact that these NCAFS subscales include multiple items involving talking to the infant. For example, contributors to higher maternal Sensitivity to Cues scores include verbally commenting on hunger, verbally commenting on satiation, and varying the intensity of verbal stimulation. Contributors to higher maternal Social–Emotional Growth Fostering scores include playing games, making positive statements, praising, and vocalizing. Contributors to higher maternal Cognitive Growth Fostering scores include verbally responding to the child, describing the feeding to the child, and talking to the child about things other than feeding. Study findings provide cross-validation of the NCAFS and suggest that maternal verbal communication during feeding is a particularly salient individual difference.

### 4.3. Suck Duration and Infant Clarity of Cues

Suck duration was negatively associated with infant Clarity of Cues. In experimental studies, when less nutrition is emitted from a nipple, suck duration shortens [41,42,43,44,45,46]. In the current study, when suck duration was shorter (i.e., less nutritive), infant Clarity of Cues subscale scores were higher. The infant Clarity of Cues subscale includes items related to the infant smiling, laughing, vocalizing, and making eye contact with the mother during feeding. Thus, infants who are more socially interactive during feeding may be sucking less nutritively. This pattern may indicate that infants are not as hungry, and therefore not sucking as nutritively and more apt to socially interact during the feeding. Alternatively, some infants may find social interaction more rewarding than feeding and therefore may be distracted from nutritive sucking.

### 4.4. Feeding Duration and Maternal Social–Emotional Growth Fostering, Maternal Cognitive Growth Fostering and Infant Responsiveness to Caregiver

Feeding duration was positively associated with maternal Social–Emotional Growth Fostering, maternal Cognitive Growth Fostering and infant Responsiveness to Caregiver. A prior study reported a positive association between feeding duration and infant Clarity of Cues [27]. Overall, it is possible that positive associations were detected because longer feedings provide more time for a range of behaviors to be exhibited. Alternatively, the feeding may last longer when mothers and infants engage in a variety of behaviors unrelated to feeding, thereby prolonging the feeding. For example, the maternal Social–Emotional Growth Fostering subscale includes items such as playing games, singing, laughing, smiling, and praising the child, while the maternal Cognitive Growth Fostering subscale includes items such as providing the child with toys or talking to the child about things unrelated to feeding and the infant Responsiveness to Caregiver scale includes items such as responding to games and social play, smiling, and vocalizing.

### 4.5. Bottle-Feeding Intensity and Infant Responsiveness to Caregiver

Bottle-feeding intensity was negatively associated with infant Responsiveness to Caregiver. The infant Responsiveness to Caregiver subscale includes items capturing when the infant responds to games, vocalizes to the caregiver, smiles at the caregiver, engages with the caregiver to start the feeding, and disengages from the feeding in the latter half of the feeding. Our findings are consistent with prior reports of bottle-feeding intensity negatively associating with NCAFS subscales [30,31,32,33], suggesting that breastfeeding may promote a more optimal mother–infant interaction, at least as captured by the NCAFS subscales. Notably, maternal Social–Emotional Growth Fostering was prospectively positively associated with breastmilk feeding intensity at 12 months, further supporting a potential bidirectional link between breastfeeding and optimal mother–infant interaction. An alternative hypothesis is that infants who are (or are perceived to be) especially voracious eaters may be more likely to be supplemented with bottle-feeding, more likely to transition more rapidly from breastfeeding to a diversified diet, and less likely to use the feeding as a time for social interaction. In other words, infants who are more focused on the task of feeding, as opposed to interacting with the caregiver, may also be more likely to be bottle-fed and to have a more expansive dietary repertoire beyond breastmilk by 12 months.

### 4.6. Null Concurrent Associations

There were no significant associations of maternal self-reported feeding behaviors, observed infant hunger cues, maternal-reported infant eating behaviors, breastmilk feeding intensity, or infant anthropometry with any NCAFS subscale concurrently. Our findings that maternal self-reported restrictive and pressuring feeding behaviors were not associated with any NCAFS subscales were not consistent with a prior report of a positive association between maternal self-reported responsive feeding style and infant Clarity of Cues [27]. This discrepancy may be due to different self-report measures of maternal feeding. Infant gaze to the bottle and fingers-to-mouth behavior in the ATDG-FIT feeding protocol did not associate with any NCAFS subscale, consistent with prior reports that fingers-to-mouth behavior may not be a robust indicator of hunger and satiety [53], and therefore perhaps not a strongly salient infant cue. It is also possible that the ATDG-FIT protocol specifically elicited these hunger cues in a manner that the typical feeding protocol (to which the NCAFS was applied) did not, because the ATDG-FIT protocol specifically required that the infant wait for the feeding. Maternal-reported infant eating behaviors were not associated with any NCAFS subscales, consistent with a prior report [27]. The null findings may be because the NCAFS subscales include a mix of feeding-promoting and -inhibiting behaviors, as well as behaviors specific- and non-specific to feeding. Alternatively, the null findings may be because the NCAFS also only captures a single feeding, while maternal report questionnaires capture infant traits exhibited over time. Like others [27], we did not detect an association with breastmilk feeding intensity in the habitual diet. Also consistent with others [28], we did not detect an association with concurrent infant anthropometry.

### 4.7. Associations of NCAFS Subscales with 12-Month Outcomes

None of the NCAFS subscales predicted food responsiveness, satiety responsiveness, food fussiness, or weight-for-length z-score at 12 months. However, maternal Social–Emotional Growth Fostering was positively associated with breastmilk feeding intensity at 12 months and maternal Sensitivity to Cues was negatively associated with rate of change in weight-for-length z-score between early infancy and age 12 months. Our results align with a prior study reporting null associations between NCAFS scores and infant weight at 6 months [28]. Results differ from a prior study that identified a negative association between infant Clarity of Cues and change in weight-for-age z-score from birth to up to 32 weeks [27] but were consistent with another study that identified a negative association between maternal Sensitivity to Cues and weight gain from ages 6 to 12 months [26]. Our results also differed from a prior study reporting a positive association between NCAFS subscales and infant weight; however, this study included only 12 infants, all of whom were underweight and may therefore not be comparable to the current cohort [25]. In summary, our differing findings for weight outcomes may be due to differences in cohort characteristics (i.e., related to baseline weight status [25] or feeding mode [27]), the manner in which weight was indexed (i.e., weight-for-age, raw weight, or categorical weight compared to weight-for-length z-score), or differences in the time frame of follow-up. Our sample size was larger than the prior studies detecting associations (i.e., *n* = 12 [25], 86 [27] and 96 [26]), making it less likely that our study was underpowered to detect effects. Our finding that more optimal maternal Sensitivity to Cues was negatively associated with change in weight-for-length z-score from early infancy to 12 months is notable. Maternal Sensitivity to Cues includes items related to the caregiver slowing the pace of the feeding in response to infant disengagement cues, only offering food when the child is attending, and terminating the feeding when the child shows satiation cues. Thus, this suggests that maternal responsiveness to the infant’s satiation cues may protect against excessive weight gain. The maternal Sensitivity to Cues subscale also includes items, however, related to the caregiver smiling with the child, making eye contact with the child, verbalizing, varying the intensity of rocking, verbal stimulation, and touching during the feeding. It is possible, therefore, that mothers who are more socially interactive with the infant during the feeding may also cause the feeding to be less nutritive, which could result in less rapid weight gain. These questions require further consideration in future work.

### 4.8. Suboptimal Internal Reliability of NCAFS Subscales

The suboptimal internal reliabilities of the NCAFS subscales deserve special mention. A major contributor to the suboptimal internal reliabilities in our sample was likely the very limited variability for many items. For example, we did not observe any episodes of making negative comments about, yelling at, roughly handling, or physically punishing the child. Mothers were generally observed to display multiple behaviors characteristic of an interaction considered to be optimal by the NCAFS, as reflected in the very high mean scores and relatively limited range for many subscales. We evaluated whether removal of certain items would improve internal reliability but did not find evidence of substantial improvement with scale modification. We did not observe a pattern that subscales with poorer internal reliability relative to others in this cohort showed fewer associations. Of note, in the NCAFS normative sample, internal consistency ranges from α = 0.56 to 0.69 across subscales [2]; the only subscale in our cohort that was consistently below this range was maternal Sensitivity to Cues, with α = 0.37 to 0.55. As has been noted by others [8], the NCAFS normative sample was coded live as opposed to from video recording (as in the current study), which is known to elevate internal consistency when the inability to recall whether an item occurred may lead to a bias to score the item like other items. Therefore, the lower internal reliability obtained in our sample may be a function of the accuracy afforded by our video recording methodology. We also note that most studies reporting on the predictive value of the NCAFS have either not tested the predictive value of the individual subscales (versus total scores) or have not reported internal reliability for the individual subscales, which prevents comparison with our results. In summary, we propose that the poor internal reliability of the NCAFS subscales is not unique to our cohort and may suggest that the subscales are not capturing coherent constructs relevant to feeding, eating, or growth outcomes. The poor internal reliabilities may also be a primary contributor to the primarily null associations identified.

### 4.9. Strengths and Limitations

Strengths included a relatively large sample, longitudinal design, and availability of multiple potential covariates. There were limitations. First, the study sample comprised healthy infants from relatively well-resourced families at low biopsychosocial risk. The low-risk sample may have reduced variability in NCAFS subscales and results may not be generalizable to dissimilar samples. Second, mothers included in the analytic sample were older than those excluded, which may have introduced selection bias. Third, infant gestational age, birth weight, and maternal pre-pregnancy weight were obtained by maternal report, which may have introduced recall bias. Fourth, there was missing data, particularly for the sucking metrics and 12-month outcomes, which may have limited power and introduced bias. Fourth, bottle-feeding may have introduced a novel feeding situation which could have changed infant behavior, though prior studies have not detected differences in NCAFS scores by feeding mode [16,54]. Fifth, we used a shortened version of the Infant Feeding Styles Questionnaire, which may have limited the ability to capture constructs related to maternal feeding at the level of detail and nuance with which the questionnaire was originally designed. Sixth, many factors that contribute to variability in infant eating behavior and weight outcomes (e.g., genetic or biological contributors) were not measured in this study and their inclusion may have improved the precision of the estimates and uncovered previously undetected associations. Seventh, the feeding was videotaped, which may have altered mothers’ behavior.

### 4.10. Implications for Utility of the NCAFS as a Measure of Maternal Feeding or Infant Eating Behaviors Relevant to Infant Growth

NCAFS subscales have been shown repeatedly to differentiate infants with socioeconomic disadvantage [6] and biopsychosocial risk [55] (i.e., prematurity [56], prenatal exposures [57], birth defects [58]) to predict cognitive performance in toddlerhood [7,9,20,21], and to correlate with observations of the home environment [2,6,7,19]. Results of the current study suggest, however, that the tool may have more modest utility in differentiating infants with regard to concurrent maternal feeding behaviors and concurrent and future infant eating behavior, diet, and growth. The primarily null associations we observed may be in part due to well-known discrepancies in observed versus reported behaviors. Alternatively, NCAFS subscales may be less sensitive to nuances of the feeding interaction because many different feeding-related constructs (e.g., both hunger and satiation in the same subscale), as well as behaviors less directly related to feeding (e.g., singing, playing, vocalizing), are included in the same NCAFS subscales. Finally, many of the NCAFS subscales showed little stability across age points, which is consistent with prior reports [2,6], but calls into question whether the maternal and infant behaviors being measured are traits or states.

## 5. Conclusions

In summary, among healthy infants, associations of NCAFS subscales with maternal feeding, infant eating, infant diet, and infant weight were primarily null. However, more maternal social interaction during feeding, less nutritive sucking, longer feeding duration, and lower bottle-feeding intensity were associated with more optimal NCAFS scores concurrently, and more optimal NCAFS scores were associated with lower weight-for-length z-score change and greater breastmilk feeding intensity prospectively. Future work should examine the many alternative hypotheses for the causes of these associations, examine prospective associations of NCAFS subscales with outcomes beyond 12 months, and consider moderators of the identified associations.

## Figures and Tables

**Table 1:** Cohort characteristics.

	Observations ^a^	Characteristic, M (SD) or *n* (%)		
Age point (mo)		0.5	2	4
Infant participants (*n*)	456	81	174	201
Infant characteristics				
Age (mo), M (SD)	456	0.87 (0.28)	2.38 (0.40)	4.49 (0.44)
Sex, *n* (%)	456			
Male		41 (51%)	85 (49%)	103 (51%)
Female		40 (49%)	89 (51%)	98 (49%)
Gestational age (wks), M (SD)	452	39.5 (1.1)	39.5 (1.1)	39.5 (1.1)
Birthweight (kg), M (SD)	440	3.4 (0.4)	3.5 (0.4)	3.5 (0.4)
Delivery mode, *n* (%)	441			
Cesarean		23 (29%)	42 (25%)	50 (26%)
Vaginal		55 (71%)	126 (75%)	145 (74%)
Birth order, *n* (%)	445			
First born		26 (32%)	51 (30%)	58 (30%)
Not first born		54 (68%)	120 (70%)	136 (70%)
Age at introduction of solids (mo), M (SD)	428	6.8 (2.3)	6.9 (2.1)	6.8 (1.9)
Race/ethnicity, *n* (%)	451			
Non-Hispanic white		43 (54%)	106 (62%)	127 (64%)
Non-Hispanic black		15 (19%)	25 (14%)	25 (13%)
Hispanic any race		6 (8%)	10 (6%)	12 (6%)
Other or >1 race		16 (20%)	31 (18%)	35 (18%)
Maternal characteristics				
Age (years), M (SD)	456	30.7 (5.1)	31.0 (5.0)	31.7 (4.9)
Education, *n* (%)	456			
≤high school diploma or GED		9 (11%)	17 (10%)	15 (7%)
Some college		30 (37%)	44 (25%)	45 (22%)
4-year college degree		22 (27%)	50 (29%)	61 (30%)
>4-year college degree		20 (25%)	63 (36%)	80 (40%)
Marital status	442			
Married		52 (66%)	124 (73%)	147 (76%)
Committed relationship		16 (20%)	23 (14%)	22 (11%)
Single, never married		11 (14%)	19 (11%)	19 (10%)
Separated or divorced		0 (0%)	4 (2%)	5 (3%)
Pre-pregnancy BMI, M (SD)	433	29.0 (8.2)	28.1 (7.3)	27.6 (7.0)
Race/ethnicity, *n* (%)	451			
Non-Hispanic white		50 (63%)	118 (69%)	144 (72%)
Non-Hispanic black		15 (19%)	24 (14%)	24 (12%)
Hispanic any race		2 (3%)	7 (4%)	7 (4%)
Other or >1 race		12 (15%)	23 (13%)	25 (12%)
Income-to-needs ratio, M (SD)	408	3.4 (2.4)	3.4 (2.1)	3.6 (2.2)
PHQ-8 Total Score, M (SD)	403 ^b^	3.86 (3.10)	3.95 (3.31)	3.79 (3.24)
NCAFS subscales				
Maternal Sensitivity to Cues, M (SD), range	456	14.2 (1.6), 10–16	13.8 (1.8), 8–16	14.2 (1.2), 9–16
Maternal Response to Distress, M (SD), range	456	10.5 (1.0), 6–11	9.8 (1.4), 5–11	9.9 (1.2), 6–11
Maternal Social–Emotional Growth Fostering, M (SD), range	456	10.8 (1.7), 6–13	10.8 (2.0), 4–14	10.8 (1.5), 4–13
Maternal Cognitive Growth Fostering, M (SD), range	456	5.5 (2.1), 1–9	5.8 (2.1), 1–9	5.7 (1.9), 1–9
Infant Clarity of Cues, M (SD), range	456	11.3 (1.9), 5–14	11.8 (1.7), 5–15	11.7 (1.8), 5–15
Infant Responsiveness to Caregiver, M (SD), range	456	5.0 (2.0), 0–9	5.8 (2.1), 2–10	5.7 (2.0), 1–9
Maternal feeding behaviors				
Proportion of ATDG-FIT delay segment that mother shows verbal communication, M (SD)	427	0.57 (0.35)	0.58 (0.32)	0.50 (0.34)
Proportion of ATDG-FIT feeding segment that mother shows verbal communication, M (SD)	427	0.21 (0.24)	0.24 (0.27)	0.19 (0.24)
IFSQ restrictive, M (SD)	448	2.62 (1.22)	2.50 (1.13)	2.67 (1.19)
IFSQ pressuring, M (SD)	448	2.06 (0.83)	1.88 (0.75)	2.03 (0.86)
Infant eating behaviors				
Proportion of ATDG-FIT delay segment that infant shows gaze to bottle, M (SD)	427	0.08 (0.18)	0.09 (0.19)	0.23 (0.30)
Proportion of ATDG-FIT delay segment that infant shows fingers to mouth, M (SD)	427	0.15 (0.20)	0.09 (0.16)	0.21 (0.25)
BEBQ enjoyment of food, M (SD)	440	4.58 (0.40)	4.54 (0.40)	4.52 (0.43)
BEBQ food responsiveness, M (SD)	436	2.61 (0.79)	2.31 (0.74)	2.16 (0.66)
Suck duration (s), M (SD)	263	0.69 (0.10)	0.67 (0.09)	0.65 (0.12)
Sucking burst duration (s), M (SD)	263	22.52 (20.19)	37.05 (36.31)	60.22 (108.79)
Sucking rate (sucks/s), M (SD)	262	1.51 (0.29)	1.56 (0.26)	1.63 (0.26)
Feeding duration (min), M (SD)	455	12.06 (7.41)	12.80 (8.12)	10.94 (7.77)
Infant habitual diet				
Breastmilk feeding intensity, M (SD)	443	0.75 (0.37)	0.74 (0.40)	0.68 (0.43)
Bottle-feeding intensity, M (SD)	436	0.47 (0.41)	0.46 (0.41)	0.52 (0.42)
Infant anthropometry				
WAZ, M (SD)	440	−0.13 (0.87)	−0.23 (0.88)	−0.10 (0.94)
LAZ, M (SD)	437	−0.32 (0.93)	−0.27 (0.92)	−0.25 (0.96)
WLZ, M (SD)	437	0.00 (0.99)	0.07 (0.98)	0.15 (0.95)
Typical feeding protocol characteristics				
Protocol bottle content, *n* (%)	448			
Breastmilk		51 (64%)	112 (65%)	113 (58%)
Formula		29 (36%)	60 (35%)	83 (42%)
Protocol milk/formula intake (g), M (SD)	450	53.17 (31.84)	66.24 (47.18)	73.54 (57.66)
Infant state at feeding initiation	456			
Quiet sleep (=1)		2 (2%)	0 (0%)	0 (0%)
Active sleep (=2)		0 (0%)	2 (1%)	0 (0%)
Drowsy (=3)		1 (1%)	2 (1%)	1 (1%)
Quiet alert (=4)		52 (64%)	99 (57%)	122 (80%)
Active alert (=5)		5 (6%)	23 (13%)	3 (2%)
Crying (=6)		21 (26%)	48 (28%)	27 (18%)
Time since prior feeding (min), M (SD)	441	136.87 (63.63)	143.11 (68.82)	160.76 (100.75)
NFANT^TM^ device present, *n* (%)	456			
Yes		45 (56%)	100 (57%)	118 (59%)
No		36 (44%)	74 (43%)	83 (41%)
		12 months		
CEBQ-T food responsiveness, M (SD)	167	2.54 (0.85)		
CEBQ-T satiety responsiveness, M (SD)	165	2.70 (0.58)		
CEBQ-T food fussiness, M (SD)	167	2.11 (0.63)		
Breastmilk feeding intensity, M (SD)	179	0.33 (0.42)		
WLZ, M (SD)	203	0.42 (0.95)		
ΔWLZ per month, M (SD)	167	0.04 (0.10)		

^a^ Represents total observations across the three age points. ^b^ Collected at age 2 months only and considered a fixed covariate for analyses. ATDG-FIT, Ability to Delay Gratification for Food in Infants Task; BEBQ, Baby Eating Behavior Questionnaire; BMI, body mass index; CEBQ-T, Children’s Eating Behavior Questionnaire-Toddler; GED, general educational development; IFSQ, Infant Feeding Styles Questionnaire; LAZ, length-for-age z-score; NCAFS, Nursing Child Assessment Feeding Scale; PHQ-8, Patient Health Questionnaire-8; WAZ, weight-for-age z-score; WLZ, weight-for-length z-score; ΔWLZ, change in weight-for-length z-score from early infancy (0.5, 2, or 4 months) to 12 months.

**Table 2:** Parameter estimates (βs) and standard errors (SEs) from unadjusted and adjusted mixed models accounting for repeated measures within subjects across age points 0.5, 2, and 4 mos predicting concurrent NCAFS subscales from maternal feeding behaviors, infant eating behaviors, infant habitual diet, and infant anthropometry.

	Maternal								Infant			
	Sensitivity to Cues		Response to Distress		Social–Emotional Growth Fostering		Cognitive Growth Fostering		Clarity of Cues		Responsiveness to Caregiver	
Model	Unadj	Adj ^b^	Unadj	Adj ^b^	Unadj	Adj ^b^	Unadj	Adj ^b^	Unadj	Adj ^b^	Unadj	Adj ^b^
Participants (*n*) ^a^	211	184	211	n/a	211	182	211	182	211	124	211	124
Observations (*n*) ^a^	426	371	426	n/a	426	358	426	362	426	238	426	234
Maternal feeding behaviors												
Proportion of ATDG-FIT delay segment that mother shows verbal communication	**0.84** (0.23) ***	0.80 (0.24) ***	0.38 (0.19)	-	**1.12** (0.26) ***	0.74 (0.29) *	**1.57** (0.30) ***	**1.05** (0.34) **	0.60 (0.28) *	-	0.80 (0.31) *	-
Proportion of ATDG-FIT feeding segment that mother shows verbal communication	0.74 (0.31) *	-	0.38 (0.25)	-	**1.39** (0.34) ***	1.10 (0.38) **	**1.92** (0.40) ***	**1.37** (0.45) **	0.80 (0.36) *	-	0.93 (0.41) *	-
IFSQ restrictive	−0.10 (0.07)	-	−0.04 (0.06)	-	−0.14 (0.08)	-	−0.12 (0.10)	-	−0.16 (0.08)	-	−0.09 (0.09)	-
IFSQ pressuring	−0.10 (0.10)	-	−0.01 (0.08)	-	−0.24 (0.11) *	-	−0.23 (0.14)	-	−0.15 (0.11)	-	−0.24 (0.13)	-
Infant eating behaviors												
Proportion of ATDG-FIT delay segment that infant shows gaze to bottle	−0.11 (0.32)	-	−0.10 (0.26)	-	−0.31 (0.35)	-	−0.55 (0.40)	-	−0.08 (0.38)	-	−0.29 (0.42)	-
Proportion of ATDG-FIT delay segment that infant shows fingers to mouth	0.22 (0.36)	-	0.31 (0.31)	-	0.28 (0.39)	-	0.72 (0.45)	-	0.21 (0.44)	-	0.54 (0.48)	-
BEBQ enjoyment of food	0.29 (0.19)	-	0.37 (0.15) *	-	0.00 (0.22)	-	−0.08 (0.25)	-	0.18 (0.22)	-	0.17 (0.25)	-
BEBQ food responsiveness	−0.15 (0.12)	-	−0.04 (0.09)	-	−0.35 (0.13) **	−0.17 (0.13)	−0.39 (0.15) *	-	−0.25 (0.13)	-	−0.21 (0.15)	-
Suck duration (s)	−1.58 (0.91)	-	−0.24 (0.77)	-	−2.12 (1.04)*	-	−1.65 (1.19)	-	**−4.35** (1.01) ***	−5.35 (1.97) **	**−3.98** (1.18) **	−1.33 (2.19)
Sucking burst duration (s) ^c^	0.15 (0.11)	-	0.21 (0.09)*	-	0.21 (0.12)	-	0.08 (0.14)	-	0.23 (0.12)	-	0.09 (0.14)	-
Sucking rate (sucks/s)	0.03 (0.37)	-	−0.06 (0.31)	-	0.16 (0.42)	-	0.30 (0.48)	-	**1.33** (0.41) **	−0.83 (0.82)	**1.51** (0.47) **	0.56 (0.93)
Feeding duration (min)	0.02 (0.01) *	-	0.00 (0.01)	-	**0.04** (0.01) ***	0.04 (0.01) ***	**0.03** (0.01) **	**0.03** **(0.01)** **	**0.05** (0.01) ***	0.01 (0.01)	**0.07** (0.01) ***	0.06 (0.01) ***
Infant habitual diet												
Breastmilk feeding intensity	0.47 (0.19) *	-	0.36 (0.15) *	-	**0.79** (0.23) ***	0.25 (0.33)	**0.82** (0.27) **	0.26 (0.40)	0.24 (0.22)	-	0.64 (0.26) *	-
Bottle-feeding intensity	−0.49 (0.19) *	-	−0.27 (0.15)	-	−0.85 (0.22) ***	−0.59 (0.32)	−0.78 (0.26) **	−0.56 (0.39)	−0.49 (0.22) *	-	−0.85 (0.25) ***	**−1.11 (0.37)** **
Infant anthropometry												
WLZ	0.04 (0.08)	-	−0.10 (0.07)	-	−0.10 (0.09)	-	−0.09 (0.11)	-	−0.06 (0.09)	-	−0.05 (0.11)	-

* *p* < 0.05, ** *p* < 0.01, *** *p* < 0.001; After applying a Bonferroni correction for multiple comparisons, covariates meeting the screening criterion (α = 0.05, corrected significance threshold *p* = 0.008) are shown in bold. ^a^ For unadjusted models, maximum sample sizes for all unadjusted models are shown; different models have different sample sizes depending on data missingness of independent variables. ^b^ Adjusted models include infant birthweight, maternal education, and maternal pre-pregnancy BMI as well as any additional covariates (including those listed in Supplementary Table S3) that met the Bonferroni-corrected screening criterion (α = 0.05, corrected significance threshold *p* = 0.008). ^c^ Log-transformed prior to analysis. ATDG-FIT, Ability to Delay Gratification for Food in Infants Task; BEBQ, Baby Eating Behavior Questionnaire; IFSQ, Infant Feeding Styles Questionnaire; NCAFS, Nursing Child Assessment Feeding Scale; WLZ, weight-for-length z-score.

**Table 3:** Parameter estimates (βs) and standard errors (SEs) from regression models predicting 12-month infant outcomes from NCAFS subscales at 0.5, 2, or 4 months ^a^.

	Children’s Eating Behavior Questionnaire-Toddler			Breastmilk Feeding Intensity	WLZ	ΔWLZ
	Food Responsiveness	Satiety Responsiveness	Food Fussiness			
Participants (*n*)	142	142	142	148	170	167
Maternal Sensitivity to Cues	0.01 (0.06)	−0.04 (0.04)	−0.01 (0.05)	0.07 (0.03) *	−0.04 (0.06)	**−0.02 (0.01)** **
Maternal Response to Distress	0.04 (0.06)	−0.09 (0.05) *	−0.08 (0.05)	0.04 (0.03)	−0.01 (0.06)	0.01 (0.01)
Maternal Social–Emotional Growth Fostering	−0.02 (0.05)	−0.03 (0.03)	0.00 (0.03)	**0.06 (0.02)** **	−0.06 (0.05)	0.00 (0.01)
Maternal Cognitive Growth Fostering	−0.02 (0.04)	0.00 (0.03)	−0.02 (0.03)	0.04 (0.02) *	−0.04 (0.04)	0.00 (0.00)
Infant Clarity of Cues	0.00 (0.04)	0.02 (0.03)	0.04 (0.03)	0.05 (0.02) *	−0.09 (0.04) *	0.01 (0.01)
Infant Responsiveness to Caregiver	−0.03 (0.04)	0.00 (0.03)	0.02 (0.03)	0.02 (0.02)	0.00 (0.04)	0.00 (0.00)

* *p* < 0.05, ** *p* < 0.01. After applying a Bonferroni correction for multiple comparisons, covariates meeting the screening criterion (α = 0.05, corrected significance threshold *p* = 0.008) are shown in bold. ^a^ Adjusted for infant age at which NCAFS obtained, infant sex, gestational age, birthweight, birth order, age at introduction of solids, maternal age, maternal education, and maternal pre-pregnancy BMI. WLZ, weight-for-length z-score; ΔWLZ, rate of change (per month) in weight-for-length z-score from early infancy (0.5, 2, or 4 months) to 12 months.

## Data Availability

Upon publication, the original data presented in the study will be made publicly available at https://deepblue.lib.umich.edu/data, accessed on 20 July 2026.

## Supplementary Materials

The following supporting information can be downloaded at https://www.mdpi.com/article/10.3390/nu18152532/s1: Table S1. Bivariate comparisons of analytic versus excluded samples; Table S2. Correlations between and within NCAFS subscales across age points; Table S3. Parameter estimates (βs) and standard errors (SEs) from unadjusted mixed models accounting for repeated measures within subjects across age points 0.5, 2, and 4 mos predicting NCAFS subscales from perinatal, demographic, and protocol characteristics.

## Abbreviations

The following abbreviations are used in this manuscript:

ATDG-FIT	Ability to Delay Gratification for Food in Infants Task
BEBQ	Baby Eating Behavior Questionnaire
BMI	body mass index
CEBQ-T	Children’s Eating Behavior Questionnaire-Toddler
d	days
IFSQ	Infant Feeding Styles Questionnaire
kg	kilograms
LAZ	length-for-age z-score
M	Mean
Min	minutes
mo	months
NCAFS	Nursing Child Assessment Feeding Scale
PHQ-8	Patient Health Questionnaire-8
SD	Standard Deviation
SE	Standard Error
wks	weeks
WAZ	weight-for-age z-score
WLZ	weight-for-length z-score
ΔWLZ	change in weight-for-length z-score
