# Supplementary material for: Primarily Null Associations Between the Nursing Child Assessment Feeding Scale and Maternal Feeding Behaviors, Infant Eating Behaviors, Diet, and Growth: A Prospective Cohort Study
Source: Nutrients. 2026 Aug 4;18(15):2532. doi: 10.3390/nu18152532 (PMC13468399; doi:10.3390/nu18152532)
Supplement: Supplementary file 1 [file nutrients-18-02532-s001.zip › nutrients-4405371-supplementary.pdf]

Table S1. Bivariate comparisons of analytic versus excluded samples.

	Analytic sample	Excluded sample	*p*-value
Participants (*n*)	227	57	
Infant characteristics			
Sex, *n* (%)			0.84
Male	115 (51%)	28 (49%)	
Female	112 (49%)	29 (51%)	
Gestational age (wks), M (SD)	39.5 (1.1)	39.5 (1.1)	0.90
Birthweight (kg), M (SD)	3.4 (0.4)	3.4 (0.4)	0.96
Delivery mode, *n* (%)			0.98
Cesarean	58 (26%)	11 (26%)	
Vaginal	162 (74%)	31 (74%)	
Birth order, *n* (%)			0.13
First born	69 (31%)	22 (42%)	
Not first born	151 (69%)	30 (58%)	
Race/ethnicity, *n* (%)			0.79
Non-Hispanic white	140 (62%)	37 (65%)	
Non-Hispanic black	29 (13%)	9 (16%)	
Hispanic any race	17 (8%)	4 (7%)	
Other or >1 race	39 (17%)	7 (12%)	
Maternal characteristics			
Age (years), M (SD)	31.5 (4.9)	29.9 (5.2)	0.04
Education, *n* (%)			0.38
≤ high school diploma or GED	19 (8%)	9 (16%)	
Some college	53 (23%)	14 (25%)	
4-year college degree	67 (30%)	15 (26%)	
>4-year college degree	88 (39%)	19 (33%)	
Marital status, *n* (%)			0.89
Married	162 (74%)	41 (79%)	
Committed relationship	28 (13%)	5 (10%)	
Single, never married	23 (10%)	5 (10%)	
Separated or divorced	6 (3%)	1 (2%)	
Pre-pregnancy BMI, M (SD)	27.8 (7.3)	27.5 (6.9)	0.79
Race/ethnicity, *n* (%)			0.66
Non-Hispanic white	158 (70%)	40 (70%)	
Non-Hispanic black	28 (12%)	10 (18%)	
Hispanic any race	10 (4%)	2 (4%)	
Other or >1 race	29 (13%)	5 (9%)	
Income-to-needs ratio, M (SD)	3.5 (2.2)	3.2 (2.2)	0.31

BMI, body mass index; GED, general educational development.

Table S2. Correlations between and within NCAFS subscales across age points.

		Maternal Sensitivity to Cues			Maternal Response to Distress			Maternal Social-Emotional Growth Fostering			Maternal Cognitive Growth Fostering			Infant Clarity of cues			Infant Responsiveness to Caregiver		
	Age point (mo)	0.5	2	4	0.5	2	4	0.5	2	4	0.5	2	4	0.5	2	4	0.5	2	4
Maternal Sensitivity to Cues	0.5																		
	2	0.21*																	
	4	0.28*	0.19*																
Maternal Response to Distress	0.5	0.05																	
	2		0.36***		0.07														
	4			−0.02	0.01	0.10													
Maternal Social-Emotional Growth Fostering	0.5	0.56***			0.04														
	2		0.56***			0.34***		0.38***											
	4			0.40***			0.08	0.27***	0.46***										
Maternal Cognitive Growth Fostering	0.5	0.67***			−0.04			0.77***											
	2		0.51***			0.31***			0.77***		0.44***								
	4			0.37***			0.07			0.69***	0.53***	0.43***							
Infant Clarity of Cues	0.5	0.34**			−0.06			0.59***			0.51***								
	2		0.39***			0.27***			0.45***			0.44***		0.20*					
	4			0.38***			−0.08			0.53***			0.39***	−0.01	0.12				
Infant Responsiveness to Caregiver	0.5	0.44***			−0.06			0.70***			0.58***			0.61***					
	2		0.46***			0.09			0.60***			0.66***			0.60***		0.44***		
	4			0.22**			−0.14*			0.67***			0.53***			0.59***	−0.04	0.17*	

* $p < 0.05$, ** $p < 0.01$, *** $p < 0.001$

Table S3. Parameter estimates (βs) and standard errors (SEs) from unadjusted mixed models accounting for repeated measures within subjects across age points 0.5, 2, and 4 mos predicting NCAFS subscales from perinatal, demographic, and protocol characteristics.

	Maternal				Infant	
	Sensitivity to Cues	Response to Distress	Social-Emotional Growth Fostering	Cognitive Growth Fostering	Clarity of Cues	Responsiveness to Caregiver
Participants (*n*)	211	211	211	211	211	211
Observations (*n*)	426	426	426	426	426	426
Infant perinatal and demographic characteristics						
Infant age (mo)	−0.11 (0.18)	−0.24 (0.15)	0.05 (0.20)	0.33 (0.23)	0.35 (0.22)	0.26 (0.24)
Infant male (vs. not)	0.05 (0.16)	−0.02 (0.13)	−0.03 (0.20)	0.14 (0.24)	0.07 (0.18)	0.14 (0.21)
Infant gestational age (wks)	0.03 (0.08)	0.04 (0.06)	0.22 (0.09)*	0.16 (0.11)	**0.22 (0.08)****	**0.31 (0.10)****
Infant birthweight (kg)	0.29 (0.20)	0.27 (0.15)	0.48 (0.25)	0.68 (0.30)*	**0.61 (0.22)****	**0.69 (0.26)****
Infant first born (vs. not)	−0.05 (0.18)	0.04 (0.14)	−0.04 (0.22)	−0.10 (0.27)	−0.03 (0.20)	−0.10 (0.24)
Maternal perinatal and demographic characteristics						
Maternal age (yrs)	0.03 (0.02)*	0.02 (0.01)	**0.06 (0.02)****	**0.07 (0.02)****	0.02 (0.02)	0.05 (0.02)*
Maternal education ≥4y college degree (vs. not)	0.34 (0.17)*	0.29 (0.13)*	**0.59 (0.21)****	0.52 (0.26)*	0.29 (0.20)	0.47 (0.23)*
Maternal pre-pregnancy BMI (kg/m^2)	0.01 (0.01)	0.00 (0.01)	0.00 (0.01)	0.01 (0.02)	0.01 (0.01)	0.01 (0.02)
Maternal PHQ-8 Total Score	−0.05 (0.04)	0.02 (0.03)	−0.08 (0.05)	−0.05 (0.05)	−0.04 (0.04)	−0.11 (0.05)*
Typical feeding protocol characteristics						
Protocol bottle content is breastmilk (vs. not)	0.27 (0.16)	0.33 (0.13)*	0.54 (0.19)**	0.56 (0.22)*	0.28 (0.19)	0.39 (0.22)
Protocol milk/formula intake (g)	0.00 (0.00)	0.00 (0.00)	0.00 (0.00)	0.00 (0.00)	0.004 (0.002)*	**0.006 (0.002)****
Infant state at protocol feeding initiation (ordinal 1-6)	0.08 (0.08)	0.03 (0.07)	0.11 (0.09)	0.17 (0.10)	**0.31 (0.10)****	0.07 (0.11)

* $p < 0.05$, ** $p < 0.01$, *** $p < 0.001$; After applying a Bonferroni correction for multiple comparisons, covariates meeting the screening criterion (α=0.05, corrected significance threshold $p = 0.008$) are shown in bold and were included in the adjusted models shown in Table 2. BMI, body mass index; PHQ-8, Patient Health Questionnaire-8.
